# Consideration of non-canonical splice sites improves gene prediction on the *Arabidopsis thaliana* Niederzenz-1 genome sequence

**DOI:** 10.1186/s13104-017-2985-y

**Published:** 2017-12-04

**Authors:** Boas Pucker, Daniela Holtgräwe, Bernd Weisshaar

**Affiliations:** Faculty of Biology & Center for Biotechnology, Bielefeld University, Bielefeld, Germany

**Keywords:** Genome annotation, Splicing, Araport11, Gene prediction hints, Reciprocal best hit

## Abstract

**Objective:**

The *Arabidopsis thaliana* Niederzenz-1 genome sequence was recently published with an ab initio gene prediction. In depth analysis of the predicted gene set revealed some errors involving genes with non-canonical splice sites in their introns. Since non-canonical splice sites are difficult to predict ab initio, we checked for options to improve the annotation by transferring annotation information from the recently released Columbia-0 reference genome sequence annotation Araport11.

**Results:**

Incorporation of hints generated from Araport11 enabled the precise prediction of non-canonical splice sites. Manual inspection of RNA-Seq read mapping and RT-PCR were applied to validate the structural annotations of non-canonical splice sites. Predictions of untranslated regions were also updated by harnessing the potential of Araport11’s information, which was generated by using high coverage RNA-Seq data. The improved gene set of the Nd-1 genome assembly (GeneSet_Nd-1_v1.1) was evaluated via comparison to the initial gene prediction (GeneSet_Nd-1_v1.0) as well as against Araport11 for the Col-0 reference genome sequence. GeneSet_Nd-1_v1.1 contains previously missed non-canonical splice sites in 1256 genes. Reciprocal best hits for 24,527 (89.4%) of all nuclear Col-0 genes against the GeneSet_Nd-1_v1.1 indicate a high gene prediction quality.

**Electronic supplementary material:**

The online version of this article (10.1186/s13104-017-2985-y) contains supplementary material, which is available to authorized users.

## Introduction

Eukaryotic genes are transcribed as a primary transcript that is subsequently converted to a mature mRNA through several processing steps including splicing. During splicing, introns [1–3] are removed from the primary transcript while exons are retained. The process is catalyzed by a RNA protein complex called a spliceosome, which exists in several variants. Based on the spliceosome variant that acts on a given intron, eukaryotic introns are classified as U2-type introns [4] that appear very frequently, or rare U12-type introns [5], respectively [6]. The highly conserved sequences at the termini of introns are not sufficient to distinguish between both types, since the U12-spliceosome can remove AT-AC introns, some other non-canonical intron variants, as well as some introns of the canonical GT-AG type [6–9]. Canonical GT-AG and non-canonical intron variants including AT-AC introns can coexist within the same gene, potentially with an effect on gene expression due to the slow removal of U12-type introns [10]. Several extremely rare terminal intron sequences were discovered and often discussed as potential artifacts, e.g. introns with GT-GG or TT-AG termini [11–14]. Further details regarding exceptional splicing events have recently been reviewed [15, 16].

Splicing processes were investigated intensively in the plant model system *Arabidopsis thaliana* [17–22], resulting in very well annotated splice sites throughout the reference genome sequence [23]. Despite attempts to annotate non-canonical splice sites automatically [24, 25], ab initio gene prediction without experimental support from e.g. RNA-Seq data (“external hints”) does not support the detection and annotation of non-canonical splice sites on genome sequence assemblies at a satisfying level [26–28]. By generating high quality gene prediction hints based on the recently released Araport11 annotation of the Col-0 sequence [29, 30], we improved the gene set generated by ab initio gene prediction based on the *A. thaliana* Niederzenz-1 (Nd-1) sequence [31].

To correlate and compare gene structures from related genomes, the first step is to define “orthologous” gene couples. Such couples can efficiently be determined by evaluating reciprocal best BLAST hits (RBHs) [32–35]. Each RBH couple consists of two genes, one from each of the two genome sequences (or genomes) to compare, which display the highest scoring hit in the other data set in a reciprocal manner [36]. RBH couples are the basis for gene-centric comparative genomics [32–35] and can also be used for synteny analysis or as guidance in a genome assembly [31].

## Main text

### Methods

#### Analysis of candidate genes

In total, 45 randomly selected Col-0 genes with non-canonical splice sites were manually inspected in a RNA-Seq read mapping produced with STAR [37] based on Araport11 data sets (listed in [30]). Reads were required to map with at least 90% of their length and 95% similarity. The number of selected cases was a compromise between the required accuracy of the results and a manageable amount for individual manual inspection. Corresponding loci in the Nd-1 sequence were identified via tblastn [38]. Gene structures around non-canonical splice sites in the Nd-1 assembly sequence [31] were annotated manually for further investigation.

Primer combinations for RT-PCR included one primer bridging an exon–exon junction with 100–500 nt distance to the other primer (Table 1). Oligonucleotides were purchased from Metabion (http://www.metabion.com/). Total RNA was isolated as described before [39]. DNAse I (M0303L, New England Biolabs) digestion was performed according to the suppliers’ protocol. cDNA synthesis was carried out using 1 µg of total RNA and ProtoScript II Reverse Transcriptase (M0368L, New England Biolabs) based on the suppliers’ protocol. Q5 High-Fidelity DNA polymerase (M0491L, New England Biolabs) was employed according to the suppliers’ recommendations (including PCR cycling conditions) for generation of amplicons. The size of the amplicons was checked by agarose gel electrophoresis. Samples were purified for sequencing by ExoSAP-IT (78201.1.ML ThermoFisher Scientific) treatment as described [40]. Sanger sequencing on ABI3730XL was applied to reveal the entire sequences as described [41]. Finally, the correct annotation of the non-canonical splice sites in the candidate genes was inspected via sequence alignments generated with MAFFT [42].

**Table 1:** The oligonucleotides listed were applied in RT-PCRs to validate non-canonical splice sites selected candidate genes in Nd-1

Name	Gene	Sequence	Length	Orientation	Recommended annealing temperature [°C]
S015	At1g79350 (*FGT1*)	GCTTCCCTGGAGTGCTGATCG	21	Forward	61
S016	At1g79350 (*FGT1*)	TCGGGTTCATCAATCGAGCATCC	23	Reverse	61
S017	At1g79350 (*FGT1*)	AAGAACAGGTAGTTTCTCCTGCTCC	25	Reverse	60
S003	At4g01800 (*AGY1*)	ACTGGTGAAGGGAAAACGCTTG	22	Forward	59
S004	At4g01800 (*AGY1*)	AATGTATATCCCGCTCAAAGGCTG	24	Reverse	59
S005	At4g01800 (*AGY1*)	TCTTCTGCTTTTCATCAACAGTGTAATG	28	Reverse	58
S018	At4g27500 (*PPI1*)	AGCCGCAGAAGGAAGAAAAGC	21	Forward	59
S019	At4g27500 (*PPI1*)	ACGCGATGAGACGAATTCCGAG	22	Forward	61
S020	At4g27500 (*PPI1*)	CTCTTGGGATCGTTTCTGGTCC	22	Reverse	59

#### Hint-based gene prediction

All representative transcript sequences of protein coding genes in the Col-0 nucleome within the Araport11 annotation, as well as the first transcripts of At4g01800 and At3g10350, were mapped to the Nd-1 genome sequence via BLAT [43]. Perl scripts provided in the AUGUSTUS package filterPSL.pl and blat2hints.pl (http://bioinf.uni-greifswald.de/augustus/binaries/scripts/) were used to convert the BLAT output into valid hints. AUGUSTUS 3.2.1 [44, 45] was run on the Nd-1 genome sequence incorporating these hints.

#### Comparison of gene predictions

Calculation of gene prediction statistics as well as comparison to the Col-0 annotation via identification of RBHs was carried out by custom Python scripts as previously described [31]. ParsEval [46] was applied to compare the GeneSet_Nd1_v1.0 and GeneSet_Nd1_v1.1 in more detail.

### Results and discussion

When analyzing the protein coding genes predicted in the recently released *A. thaliana* Nd-1 genome sequence [31], we observed complete absence of introns with non-canonical splice sites in the initially predicted gene set (GeneSet_Nd-1_v1.0). The structural annotation was performed ab initio using AUGUSTUS 3.2. By comparing the GeneSet_Nd-1_v1.0 with the Araport11 gene set for the Col-0 reference genome sequence [23, 29, 30], we identified several loci with gene structures showing mis-annotated introns or even a lack of gene prediction for the Nd-1 case. For the present study, we focused on protein encoding genes in the nuclear genome sequence since this gene set was previously predicted ab inito. The annotation update provided here will further support *A. thaliana* pan-genomic research by redefining the gene set for the accession Nd-1. Moreover, researchers interested in single genes and their Nd-1 alleles will be able to access a high quality annotation for comparison to Araport11 for the Col-0 reference sequence.

**Fig. 1:**
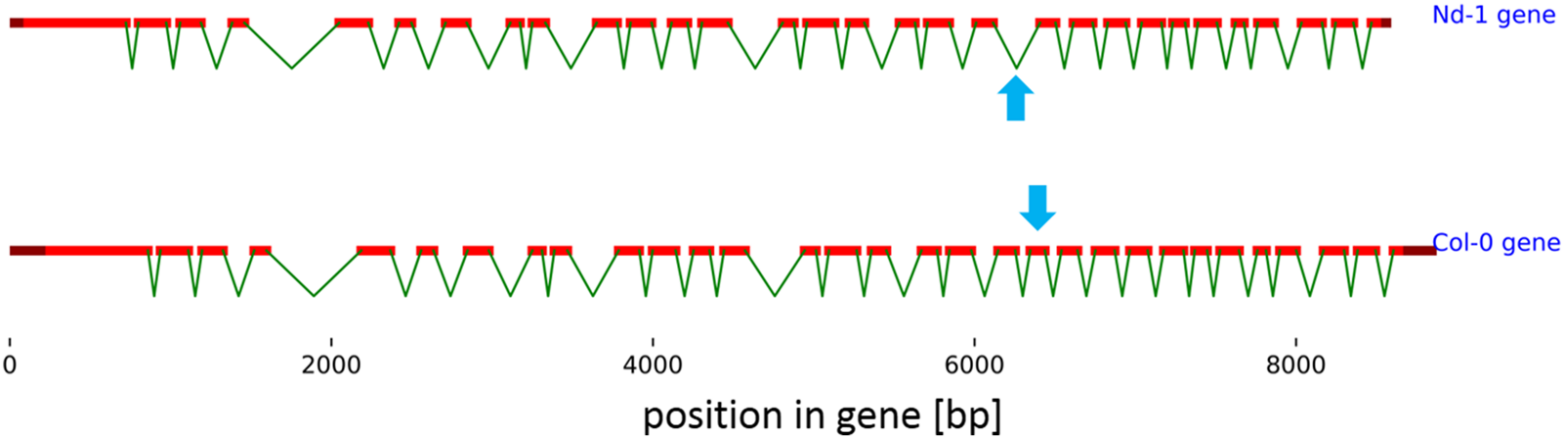
Representative gene structure of missed non-canonical splice sites in ab initio gene prediction on the Nd-1 genome sequence. Gene structures of *At1g79350*.1 and the corresponding reciprocal best BLAST hit (RBH) of the ab initio gene prediction in Nd-1 (GeneSet_Nd-1_v1.0) are displayed. The non-canonical splice sites were missed leading to a difference at exon 20 (blue arrows). Despite this deviation, the structure of *At1g79350* ^Nd−1^ was predicted very well by AUGUSTUS [44, 45]

In total, the Araport11 gene set contains 1267 genes which display non-canonical splice sites to generate the respective representative transcript. This ‘representative transcript’ has been defined as the transcript isoform containing the longest protein coding sequence (CDS) [30]. We established a set of well investigated genes consisting of At1g79350 (*FGT1*) [47–49], At4g01800 (*AGY1*) [47, 50–52] and At4g27500 (*PPI1*) [53–57] as examples for genes containing confirmed introns with non-canonical slice sites in their main transcript isoform. Despite high sequence conservation between Col-0 and Nd-1, the gene structures predicted at these loci in GeneSet_Nd-1_v1.0 did not match the Araport11 annotation [29, 30], indicating that *bona fide* genes were missed by ab initio annotation of the Nd-1 genome sequence because they contain introns with non-canonical splice sites (Fig. 1).

When analyzing the Araport11 data set of Col-0 protein coding nuclear genes, which is based on very high coverage RNA-Seq information, we identified 39 different pairs of splice donor and splice acceptor sites (i.e. intron types) that need removal in order to generate the representative transcript isoforms. In total, the Araport11 structural annotation dataset contains 119,097 splice site pairs (introns) in nuclear protein coding genes that are spliced out of the primary transcript to produce the representative transcript. Of these, 117,732 (98.9%) were canonical GT-AG splice site pairs, while 1196 (1.0%) were GC-AG pairs and 81 (0.1%) were AT-AC pairs. In addition, diverse and less frequent splice site pairs sum up to 88 (0.1%) cases. These less frequent splice site pairs occur with very low frequencies and case numbers between one and nine.

When considering all transcript isoforms of all genes annotated in Araport11, 125 different splice site pairs are annotated. Obviously, non-protein coding genes contribute a huge proportion to splice site variation. Despite the very high quality of the *A. thaliana* Col-0 reference sequence, sequencing errors or collapsed gene sequences [58] could explain at least a fraction of the very rare splice site pairs [11].

Representative structures of protein encoding genes from Araport11 were used to produce gene prediction hints for the Nd-1 genome sequence (see "Methods"). This information transfer was done to harness the improvement potential of 1267 annotated protein encoding genes in the Col-0 reference sequence containing various non-canonical splice sites in their representative transcript. Gene prediction on the Nd-1 genome sequence using these hints revealed 30,834 genes (GeneSet_Nd-1_v1.1, Additional file 1) exceeding the number of predicted genes in the GeneSet_Nd-1_v1.0 by 2164. Detailed comparison revealed a match of 91.2% in respect to predicted CDS features and a match of 50.2% concerning UTR features, respectively. Vast changes in the UTR prediction could be explained by the incorporated hints, since the ab initio prediction of these regions is error-prone. A slight reduction in the average CDS length from 1086 bp (median) in the GeneSet_Nd-1_v1.0 compared to an average length of 1041 bp (median) in the GeneSet_Nd-1_v1.1 was observed. There are 135,356 introns with 30 different pairs of donor and acceptor splice sites in the GeneSet_Nd-1_v1.1 (Additional file 2), supporting the assumption that some minor splice sites in the Araport11 annotation might be due to sequencing errors [11]. Splice site pairs were distinguished into 134,004 (99.0%) GT-AG splice site pairs, 1080 (0.8%) GC-AG splice site pairs, 66 (0.05%) AT-AC splice site pairs and 206 (0.15%) diverse and less frequent splice site pairs. In total, 1256 genes within the GeneSet_Nd-1_v1.1 contain introns with non-canonical splice sites. Their average transcript length is 2003 bp (median) consisting on average of ten protein encoding exons. Compared to the average number of four annotated exons in all genes of GeneSet_Nd-1_v1.1, we see a clear accumulation of non-canonical splice sites in exon-rich transcripts. This overrepresentation of exon-rich transcripts among the non-canonically spliced transcripts is supported by the Araport11 annotation where the average exon number of protein encoding transcripts with non-canonical splice sites is also ten. Manual inspection identified At4g01800 and At3g10350 as genes where the representative transcript in Araport11 does not require processing of non-canonical splice site pair, but another strongly expressed isoform does. Therefore, we expect the number of genes with non-canonical splice sites in Col-0 to be slightly higher than 1267 as deduced from the representative transcript data set.

Reciprocal best BLAST hit (RBH)-based comparison of the new GeneSet_Nd1_v1.1 and the Araport11 annotation revealed 24,527 gene couples (Additional file 3). The number of RBHs within the hint-based GeneSet_Nd1_v1.1 is strongly increased compared to the ab initio predicted GeneSet_Nd1_v1.0. We expect a further increase in prediction accuracy if the underlying sequence would be available with enhanced continuity, as for example possible if generated by SMRT sequencing, and if incorporation of additional hints from RNA-Seq data would be possible. High sensitivity mapping of Col-0 exon sequences to the Nd-1 genome sequence might discover small matches leading to further prediction improvements. Gene duplications are a special challenge in this process, because exon sequences might map to only one copy in the Nd-1 genome sequence. This might explain a part of the observed difference between the Col-0 annotation and the Nd-1 gene prediction concerning the number of transcripts with non-canonical splice sites.

**Fig. 2:**
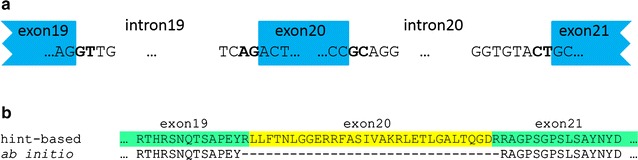
Representative gene structure of missed non-canonical splice sites in ab initio gene prediction in Nd-1. Gene structure of the At1g79350 RBH in the hint-based gene prediction (GeneSet_Nd-1_v1.1) on the Nd-1 genome sequence is displayed (**a**). The non-canonical splice sites were missed in the ab initio gene prediction leading to a skipping of exon 20 (highlighted in yellow) (**b**)

Non-canonical splice sites in the reciprocal best hits (RBHs) of the three candidate genes *FGT1*, *AGY1* and *PPI1* in the GeneSet_Nd1_v1.1 were confirmed by Sanger sequencing of amplicons generated from cDNA. *FGT1* contained 31 exons and displayed a GC-CT splice site pair in intron 20 (Fig. 2). *AGY1* contained 20 exons and displayed a GA-AG splice site pair in intron 4. *PPI1* contained 7 exons and displayed a GA-AG splice site pair in intron 6.

## Limitations

Allowing an increased number of alternative splicing possibilities deviating from the GT-AG rule would render ab initio prediction of gene structures almost impossible. Since the number of non-canonical splice sites is low, the ratio of false positive predictions would strongly increase. Incorporation of evidence from RNA-Seq experiments or high quality annotations of related genome sequences into a gene prediction process with AUGUSTUS [44, 45] or a combination of AUGUSTUS and GeneMark [59] within BRAKER1 [60] is most probably the best way to achieve high quality gene predictions. Annotating new genome sequences via transfer of annotations from model species and adding additional expression data derived hints was successfully carried out several times before and has recovered many non-canonical splice sites [61–65]. Other promising approaches are completely based on homology to predict gene structures [66]. Nevertheless, the accurate prediction of non-canonical splice sites remains a challenge. Anyway, it will be a general contribution to accuracy to pay attention to non-canonical splice sites when applying ab initio gene prediction.

## Additional files

**Additional file 1.** GeneSet_Nd1_v1.1. Gene prediction of the Nd-1 genome sequence containing genes with non-canonical splice sites.

**Additional file 2.** Non-canonical splice sites in Nd-1. All occurrences of the different splice site pairs within the first transcript of predicted Nd-1 genes in GeneSet_Nd1_v.1.1 are listed.

**Additional file 3.** Reciprocal Best Hits. Gene couples with reciprocal best hits between the Araport11 annotation of Col-0 and the GeneSet_Nd1_v1.1 are listed.
